# Detection of Aircraft Touchdown Using Longitudinal Acceleration and Continuous Wavelet Transformation

**DOI:** 10.3390/s20247231

**Published:** 2020-12-17

**Authors:** Jerzy Bakunowicz, Paweł Rzucidło

**Affiliations:** 1 Frenchay Campus, The University of the West of England, Coldharbour Lane, Bristol BS16 1QY, UK; jerzy.bakunowicz@uwe.ac.uk; 2 Department of Avionics and Control Systems, Faculty of Mechanical Engineering and Aeronautics, Rzeszów University of Technology, al. Powstańców Warszawy 12, 35-959 Rzeszów, Poland

**Keywords:** virtual sensing, touchdown, detection, aeroplane, light aircraft, landing gear, continuous wavelet, accelerometers, acceleration, MEMS

## Abstract

The paper presents a methodology enabling the detection of aircraft touchdowns based on data obtained from accelerometers attached to the structural parts of the airframe in the cockpit or passenger compartment. Precise determination of the moment and place of touchdown of the main landing gear is challenging when analysing parameters such as height, flight speed and rate of descent. During the tests of the I-31T aircraft, it turned out that vibrations of the aircraft structure caused by the contact of the front and main landing gear with the ground have a repetitive character. In particular, this applies to longitudinal acceleration. The use of continuous wavelet analysis (CWT) allowed finding unique periodic features of the landing phenomenon that distinguish it from other forms of vibration occurring in individual flight phases. Ground and flight observations of experimental aeroplane MP-02 Czajka verified the proposed method of virtual touchdown detection. The results presented in this paper justify that this method may find broader application, especially for the light aircraft class.

## 1. Introduction

The unambiguous set down of a touchdown point and time of the landing seems to be a relatively simple task when observing the process from outside of the aeroplane at a fixed position. It becomes manageable even more when using dedicated optical and recording devices. However, the process becomes complicated if the measurement takes place directly on board of a commercially operating aeroplane or, ultimately, when analysing data collected from flight recorders in the paper [1] touchdown point detection of an A320 airliner is determined based on comparison of data recorded from two radio altimeters and an accelerometer. Small aeroplanes are not commonly equipped with specialized sensors and most of the recorded data onboard comes from Global Navigation Satellite System (GNSS) receivers. On the basis of such data, it is only possible to determine the approximate landing site [2]. An additional complication comes with the required accuracy both in space and time domains. Furthermore, the limited number and quality of data available render the task challenging. The undercarriages of transport and corporate aeroplanes possess weight on wheels (WOW) sensors, which provide information about the touchdown of a particular leg. There are almost no such solutions in normally operated sport, tourist aeroplanes and gliders. Experimental application of dynamometer sensors in a PZL-104 Wilga aeroplane is presented in paper [3]. The same team presented advanced research with the use of GNSS/INS (inertial navigation system) and an optical sensor for determining general aviation (GA) aeroplane take-off and landing performance on a grassy airfield in the work [4]. The use of onboard cameras and image analysis systems may in the future enable precise measurements for the needs of normal operation of small aviation, but such works are currently at an experimental level [5].

The unmistakable determination of the moment when the wheels contact the runway surface is crucial for automatic control systems and active dumpers [6]. The signal enables effective braking of the aircraft. It may also increase the crew situational awareness, such as the warning and alerting system that braking is not possible under given meteorological conditions, for example, in Runway Overrun Prevention System (ROPS). The exact determination of the touchdown location is of interest to the manufacturers of transport aircraft. The precise detection of the touchdown location and time is not trivial, according to measurements performed on the A350 aeroplane [7].

Designers and operators of remotely controlled aerial vehicles face similar problems. Information about the contact between the aircraft undercarriage and the ground may be necessary to change the operating mode of the control system or to adapt the operating parameters of the control laws. The stringent restrictions on the dimensions and masses of unmanned aircraft mean that the installation of measuring systems (e.g., miniature WOW equivalents) is often out of the question. In these cases, a virtual sensor seems to be a sensible solution. They can be defined as sensors measuring at a location where the sensor should physically be placed, but it is not. Examples of such solutions from various fields are presented in the works [8,9,10]. Virtual measurement methods enable the determination of specific quantities based on measurements from other physically available sensors. This work focuses on this class of sensor solutions, and the motivation for it were the results obtained during flight tests of an experimental light turbine-powered aircraft I-31T [11].

## 2. The Idea of a Touch-Down Virtual Sensing

### 2.1. Genesis

**Figure 1:**
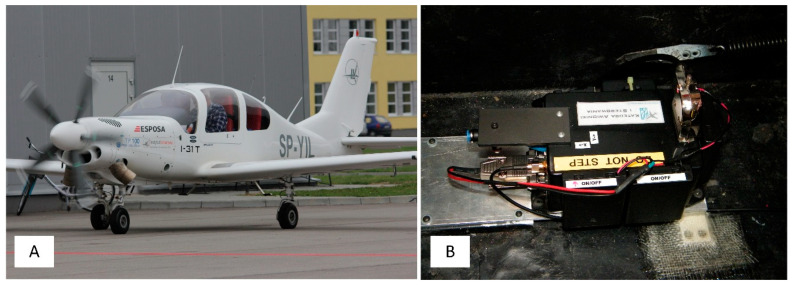
I-31T aeroplane (**A**) and flight data measurement system (**B**).

**Figure 2:**
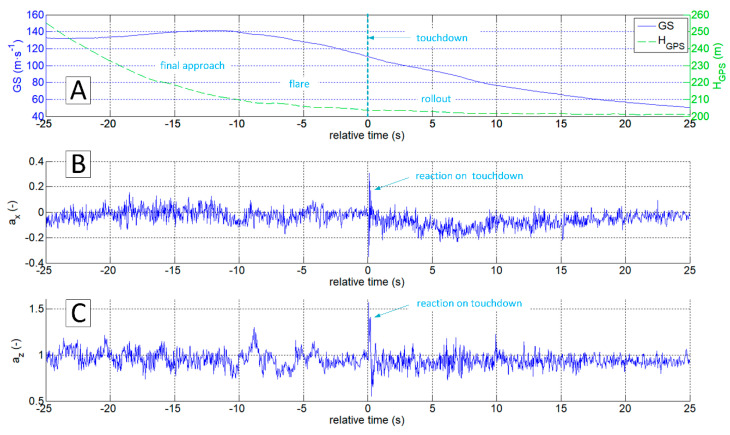
Time plots of GS and H_GPS_ (**A**), a_x_ (**B**) and a_z_ (**C**) during landing; flight No.03, ESPOSA.

**Figure 3:**
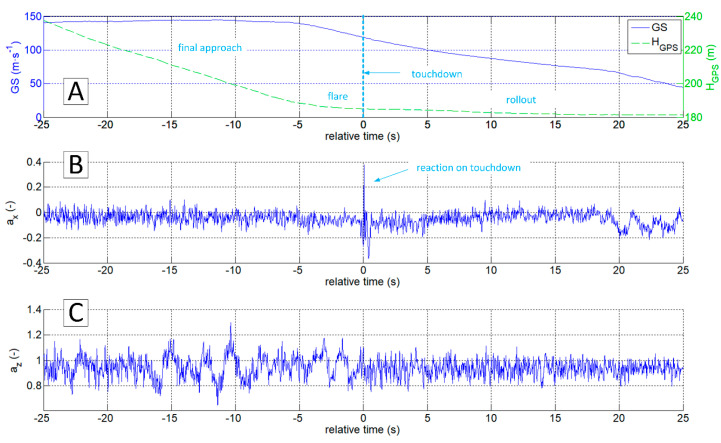
Time plots of GS and H_GPS_ (**A**), a_x_ (**B**) and a_z_ (**C**) during landing; flight No. 07, ESPOSA.

The authors of this work have encountered the problem of determining the exact moment and touchdown point by analysing data gathered during the test campaign of the I-31T turboprop experimental light aeroplane in the Efficient Systems and Propulsion for Small Aircraft (ESPOSA) project [11]—Figure 1A. During one of the landings, the pilot unexpectedly experienced the shimmy vibrations of the front landing gear. The authors presented the analysis of this occurrence in detail in the 2017 paper [12]. One of the problems identified that required solution was the determination of the exact time of a touchdown by airframe vibrations analysis. The information about altitude and speeds is, obviously, not sufficient as presented in Figure 2A and Figure 3A. Depending on the wind speed and direction, touchdown may occur over a relatively wide range of speeds, having only the speed relative to the ground in the available data sets.

On the other hand, the information on airspeed only gives a general impression on the landing process. Most runways and landing sites are not perfectly flat and level. The differences between the extremes of the runway may reach up to several tens of meters. Another problem associated with the detection of a touchdown point in a classic approach is the height or altitude measurement. In this case, barometric and satellite measurement methods are not sufficiently precise in the time and space domain, as discussed in [13].

The task in question required a solution based on data recorded by the autonomous flight parameter recorder on board the I-31T aeroplane [12,14]. There was no connection between the device (Figure 1B) and onboard installations. It also had an independent power supply and GNSS antenna system [15,16]. The system recorded the following data:

- a_x_, a_y_, a_z_ accelerations; sampling frequency 50 Hz,
- angular rates p, q, r; sampling frequency 50 Hz,
- Euler angles Φ, Θ, Ψ, sampling frequency 50 Hz,
- static pressure inside the non-pressurised cockpit p_s_; sampling frequency 10 Hz
- navigation data ϕ_GPS_, λ_GPS_, H_GPS_, TT, GS (latitude, longitude, altitude, true track, and ground speed); sampling frequency 10 Hz,
- Global Positioning System (GPS) time; sampling frequency 10 Hz.

### 2.2. Continuous Wavelet Transformation (CWT)

**Figure 4:**
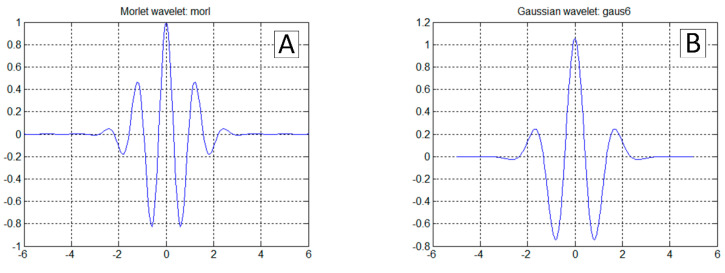
Example of Morlet wavelet (**A**) and Gaussian wavelet (**B**).

The most straightforward answer to the research question would seem to be the analysis of $a_{z}$ acceleration time series, because its rapid increase should be expected at the time of a touchdown (Figure 2C). This approach is correct in general. However, it is not sufficient because the touchdown process can be carried out smoothly, without an unmistakable accent (Figure 3C). On the other hand, the acceleration $a_{x}\;$ takes the negative value when the tyre touches the runway surface. Therefore the analysis of the $a_{x}$ looks promising (Figure 2B and Figure 3B). Unfortunately, $a_{x}$ was not measured directly on the undercarriage leg. A set of micro-electro-mechanical system (MEMS) accelerometers [17] was attached to the floor in the cockpit, close to the centre of gravity. The incipient touchdown effect manifests as vibrations transmitted by the aircraft’s structure to the sensors. Direct interpretation of raw $a_{x}$ and $a_{z}$ time-series might introduce significant inaccuracies, as in [7,12]. The characteristic phenomenon accompanying the touchdown of the landing gear (both main and front in the case of I-31T) is the formation of longitudinal oscillations (recorded mainly in the form of $a_{x}$ changes; Figure 2B and Figure 3B). These oscillations are visible if the selected time series of recorded parameters are processed with continuous wavelet analysis (CWT). The authors applied Morlet wavelet as the basic wavelet (Figure 4A). This wavelet enables precise location during shock, seismic, short-term and highly damped oscillations [18].

The continuous wavelet transformation of the function f(t) is determined by Equation (1). The function $\psi_{\mathrm{ab}}{}^{*}\left(t\right)$ is the basic wavelet and is described by Equation (2). The parameter a is a scale, while b is a wavelet shift.

(1) $$\mathrm{CWT}\left(a,b\right)=\int_{-\infty }^{+\infty }f\left(t\right)\cdot \psi_{\mathrm{ab}}{}^{*}\left(t\right)\mathrm{dt}.$$

(2) $$\psi_{\mathrm{ab}}{}^{*}\left(t\right)=\frac{1}{\sqrt{a}}\psi \left(\frac{t-b}{a}\right),$$

where ψ(t) is a continuous function called the mother wavelet.

The normalisation $1/\sqrt{a}$ in the equation ensures that the integral energy given by each translated and dilated wavelet is independent of the scale parameter a [19]. It also guarantees that the wavelet transform at each scale a is comparable directly to each other and to the transforms of different time series [20]. The mother wavelet should satisfy the so-called, admissibility condition given by Equation (3).

(3) $$\int_{-\infty }^{\infty }\frac{{\left|\Psi \left(f\right)\right|}^{2}}{\left|f\right|}\mathrm{df}<\infty ,$$

where Ψ(f)— Fourier transform of ψ(t).

Fourier transform and its modifications are base on harmonic waves, while the functions in CWT are families of wavelets (e.g., Morlet coarse wavelets (Figure 4A), Gauss (Figure 4B), Mexican Hat, Meyer’s infinite regular wavelets, Daubechies orthogonal wavelets and compact bi-wavelets based on B-splines). CWT transform enables the full reconstruction of function f(t) by inverse transform (4).

(4) $$f\left(t\right)=\frac{1}{C_{\psi }}\int_{-\infty }^{+\infty }\int_{-\infty }^{+\infty }\mathrm{CWT}\left(a,b\right)\psi_{\mathrm{ab}}{}^{*}\left(t\right)\frac{\mathrm{da}\cdot \mathrm{db}}{a^{2}}.$$

**Figure 5:**
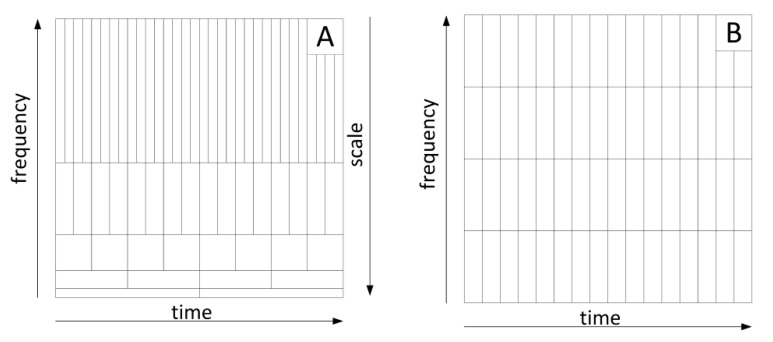
Symbolic division of the time-frequency plane in the wavelet transform (**A**) and for comparison in the short-time Fourier transform (STFT) (**B**).

Continuous wavelet analysis enables precise location in time of fast-changing phenomena. Decreasing the scale a increases the frequency carrier of the wavelet, but this is connected to the simultaneous increase of its time carrier. The resolution of the transformation relative to time and frequency is dependent on the scale factor a. As a result of the scaling process, the time and frequency are divided schematically in Figure 5A. According to the Heisenberg ambiguity principle, it is not possible to obtain any resolution in the time and frequency domain simultaneously.

Wavelet analysis gives better frequency resolution and worse time resolution for large scale values. For small scale values a we have the opposite situation. For comparison, in short time Fourier transformation (STFT), the resolution in the time and frequency domain is constant, making it difficult in many cases to isolate short-term processes (Figure 5B).

### 2.3. Proposed Method and Algorithm

**Figure 6:**
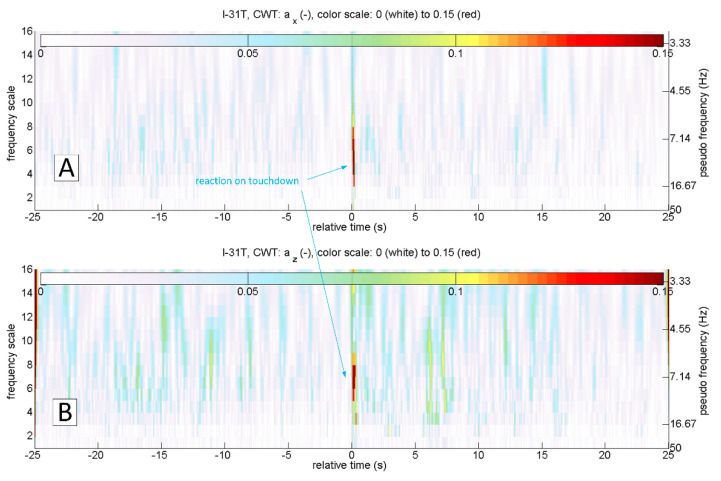
Continuous wavelet transform of a_x_ (**A**) and a_z_ (**B**); flight No. 03, ESPOSA.

**Figure 7:**
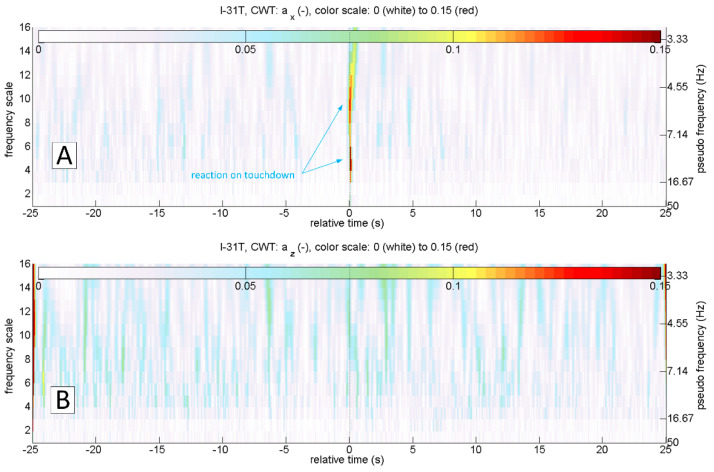
Continuous wavelet transform of a_x_ (**A**) and a_z_ (**B**); flight No. 07, ESPOSA.

**Figure 8:**
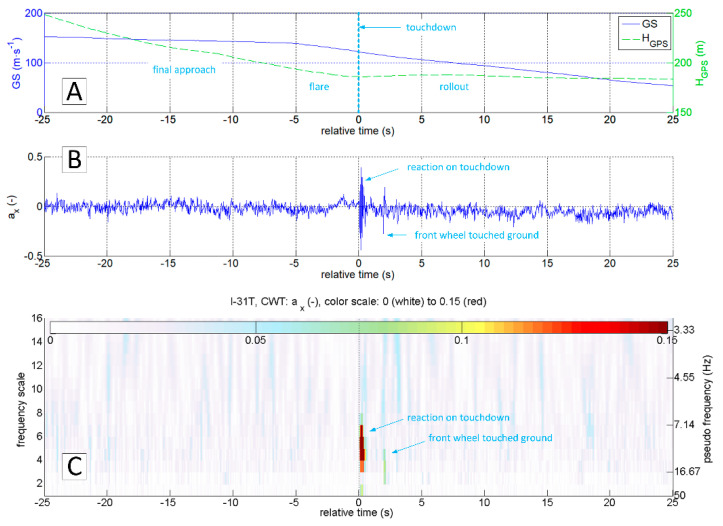
Time plots of GS, H_GPS_ (**A**), a_x_ (**B**) and continuous wavelet transform of a_x_ (**C**); flight No. 01b, ESPOSA.

Figure 6 shows the results of continuous wavelet transform of $a_{x}$ and $a_{z}$ accelerations in a 50 s sequence. The touchdown occurred after 25 s relative to the starting time from the beginning of the presented sequence. This landing was characterised by an evident touchdown of the main landing gear, visible on the CWT scalograms of the $a_{x}$ and $a_{z}$ variables. As mentioned earlier, touchdown detection based on the analysis of the $a_{z}$ variable will not always be useful. In the case of a smooth and delicate touchdown, the vibrations along this axis will have negligibly small amplitude. An example of such a landing is presented in Figure 7. Continuous wavelet analysis enables accurate touchdown detection, perfectly visible on the $a_{x}$ plot (Figure 7A). The amplitude of $a_{z}$ vibrations, in this case, is comparable to the amplitude of vibrations coming from other sources (Figure 7B)—such as the engine or the rolling of the plane on the runway surface. In both cases presented, the landing might have looked like the front gear touched the runway almost precisely at the same time as the main (so-called three points landing). Alternatively, the aeroplane’s nose lowered very gently. Therefore the front wheel impact on the runway is barely visible. In many other flights, it was possible to identify the moment when the aeroplane’s nose lowered, and the front wheel touched the ground. An example of such a landing is shown in Figure 8. Vibrations along the x-axis, associated with the contact of individual wheels (main landing gear and front wheel), are visible both on the time series of $a_{x}$ and the corresponding CWT transform. Interestingly, this landing also did not mark any changes in the course of $a_{z}$ and the corresponding CWT scalogram. Practical absence of $a_{x}$ oscillations during the flight (in particular in the range of the frequencies analysed) becomes an additional argument in favour of using acceleration analysis in the longitudinal axis to identify the touchdown of the aircraft. The oscillations of the variable $a_{z}$ in flight are visible in Figure 2C, Figure 3C, Figure 6B and Figure 7B.

Therefore, the reader may ask the question: “what is the point of using CWT since one can easily recognise the touchdown phenomenon on $a_{x}$ time histories?” However, the CWT charts allow precise location of the phenomenon in time, with accuracy close to the resolution of the transform. The touchdown of the aeroplane causes a rapid change in the acceleration $a_{x}$. A step function can be mapped using an infinite number of harmonic components. Assuming such nature of the $a_{x}$ during touchdown, one can expect its trace in all frequency components of the signal. The CWT resolution for scale equal to 1 is close to the Nyquist frequency (limit frequency) of the discrete signal. Finding the phenomenon trace for smaller scales allows for much more accurate time analysis of CWT (Figure 5A) than in methods based on Fourier transform, in which the accuracy of location of phenomena in time is constant, independent of the signal frequency and equal to the width of the analysing window (Figure 5B).

**Figure 9:**
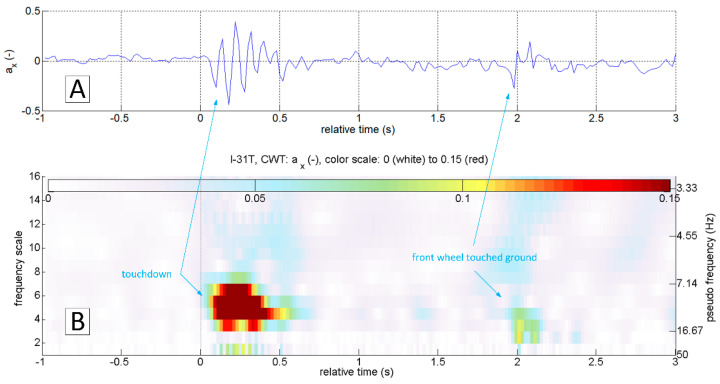
Accurate location of the touchdown based on the value a_x_ (**A**) and continuous wavelet transform (**B**); flight No. 01b, ESPOSA.

Interpretation of time series can be cumbersome and requires an individual approach and assessment of the phenomenon. The ability to assess not only the amplitude but also the frequency of structural vibrations occurring during the landing of the aircraft immediately (free vibrations and vibrations coming from the ground after the touchdown) favours using a continuous wavelet transform. These frequencies are in the range of 5–16 Hz for the I-31T aeroplane (Figure 6, Figure 7 and Figure 8). Figure 8 shows a typical landing. The touchdown of the front wheel followed touchdown of the main landing gear, and both are visible on plots. In Figure 9, a detailed analysis of this process was carried out for 4 s. Oscillations of $a_{x}$ acceleration related to the touchdown of the main landing gear are relatively short in duration, extinguishing very rapidly after 0.5 s. This promises a good starting point for a virtual sensor characterised by quick response time.

Touchdown detection directly based on the values of the wavelet transform coefficients of the acceleration $a_{x}$ requires analysis of the obtained scalograms. In practice, it could be reduced to a qualitative visual interpretation of the image, as mentioned above. However, it is possible to develop functions that implement automatic touchdown detection in the analysed data sets. The authors focused on relationships that allow determining the moment of touchdown based on the recorded acceleration values $a_{x}$ only. Other flight parameters, if necessary, can provide additional support for the method. The verification of the technique used three different virtual signals (VSig).

The first virtual signal (VSig_1_), described by the Formula (5), assumes that extreme CWT coefficients will occur at the time of landing. The maximum values sought were divided into two ranges: 1–31 and 32–256, respectively. By observing a series of landings, it was established (Table 1) that the weight of the components with frequencies corresponding to the 32–256 scales is much higher than for lower scale values. The CWT coefficient reached its maximum value in the range 1–31 in landing No. 15 only. Therefore, an empirical weight factor of 4 was adopted in formula:

(5) $${\mathrm{VSig}}_{1}\left(b\right)=\underset{a\in <1–31>}{\max}\mathrm{CWT}\left(a,b\right)+\underset{a\in <32–256>}{\max}4\cdot \mathrm{CWT}\left(a,b\right).$$

The second relationship (6), as well as the third (7), assumes that during the touchdown, the sum of the scale factor values will reach the local extreme.

(6) $${\mathrm{VSig}}_{2}\left(b\right)=0.512\cdot \overset{256}{\underset{a=32}{\sum }}\mathrm{CWT}\left(a,b\right).$$

(7) $${\mathrm{VSig}}_{3}\left(b\right)=0.373\cdot \overset{256}{\underset{a=1}{\sum }}\mathrm{CWT}\left(a,b\right).$$

Formula (6) takes into account the sum of CWT for the 32–256 scale range, while Formula (7) takes into account the entire range of frequencies. The scaling coefficients 0.512 and 0.373 have been chosen deliberately, so that the standard deviations of noise (σ_in_) of the virtual signals (6) and (7) have the same values with VSig_1_ (5). The coefficients were calculated using the measurement database from research flights of I-31T. To determine whether a touchdown has been detected at a given time, the relationship (8) should be used, which compares the values returned by the virtual signals with the sensitivity threshold.

(8) $$\mathrm{DETECTION}\;\left(b\right)=\begin{array}{c} 1{\;\mathrm{if}\;\mathrm{VSig}}_{i}\left(b\right)\geq {\mathrm{Thr}}_{i}\\ 0\;\mathrm{otherwise} \end{array}.$$

The threshold values enable detection of the smoothest landing, because of the signal to noise ratio (SNR) level. Incomplete landing No. 5 (short touchdown on one wheel followed by climbs) was excluded from the analyses (Table 1). The individual SNR values for virtual signals were determined using the measurement data and according to the dependency [21]:

(9) $${\mathrm{SNR}}_{i}=\frac{A_{i}}{\sigma_{\mathrm{in}}}.$$

Calculated values of SNR for VSig_1_–VSig_3_ are: SNR_1_ = 9.34 dB, SNR_2_ = 12.19 dB and SNR_3_ = 10.59 dB. Detection thresholds were determined reducing the value of virtual signal weakest peak (A_i_) by 10% and rounding to the nearest tenth: Thr_1_ = 0.8, Thr_2_ = 1.5 and Thr_3_ = 1.

## 3. Verification of the Methodology

### 3.1. Testing Platform

**Figure 10:**
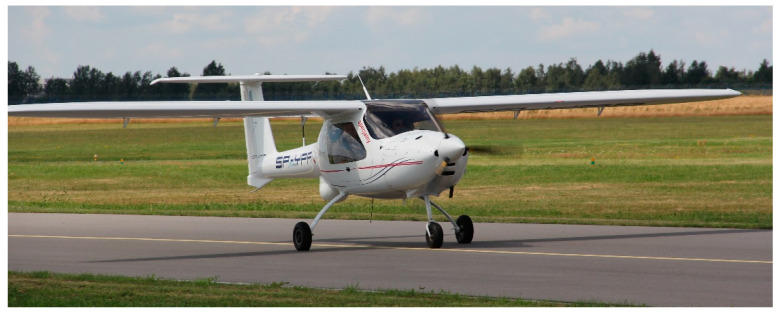
The experimental MP-02b “Czajka” aeroplane.

A different experimental aeroplane was used to verify the methodology; a flying laboratory based on a serial ultralight design MP-02 Czajka (Figure 10). It was equipped with a control system implemented as part of the terrain flying surveyor (LOT—latający obserwator terenu in Polish) project [22]. The avionics installed onboard included, among others, integrated Dynon Avionics D700. The system allows recording of many flight parameters, navigation data as well as the parameters of the engine. The sampling rates are 4 Hz in the emergency flight recorder mode and up to 16 Hz in the user-programmed mode. 

**Figure 11:**
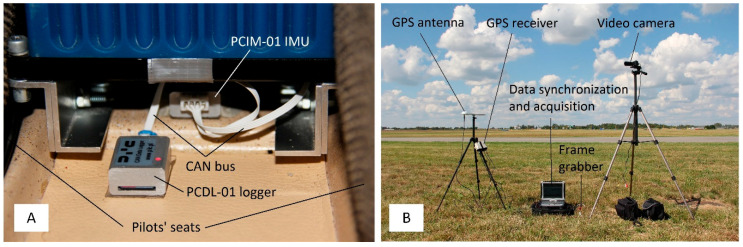
Flight data measurement system (**A**) and ground survey system (**B**).

In contrast to the data sets obtained during the I-31T tests, the data from the D700 on MP-02 recorder include information on indicated air speed (IAS), true air speed (TAS) and engine revolutions per minute (RPMs). Unfortunately, the $a_{z}$ acceleration has a quantisation level not higher than 0.1, which, in practice, makes it impossible to analyse this parameter quantitatively. The D700 system does not allow the acquisition of $a_{x}$ acceleration. Additional, custom made, onboard equipment includes the miniature PCDL-01 recorder (Figure 11) and a network of small measuring modules [23], in particular:

- PCDA-01 air data computer;
- satellite navigation receiver PCGP-01;
- PCIM-01 inertial measurement system.

The devices operate in the CANaerospace standard [24,25], enabling measurement and recording of inertial quantities with a frequency of 1 kHz. Navigation data is acquired and recorded at a frequency of 10 Hz, while aerometric data is at a frequency of 100 Hz. Both the D700 and PCDL-01 systems allow synchronisation of recorded data with the standard GPS time. In practice, this means the possibility of mutual synchronisation of data sets obtained from two independent measurement and recording systems. The D700 system has 1/16 of a second accuracy. The PCDL-01 and PCGP-01 systems have been designed to achieve accuracy in synchronisation with a GPS time standard close to 0.001 s.

**Figure 12:**
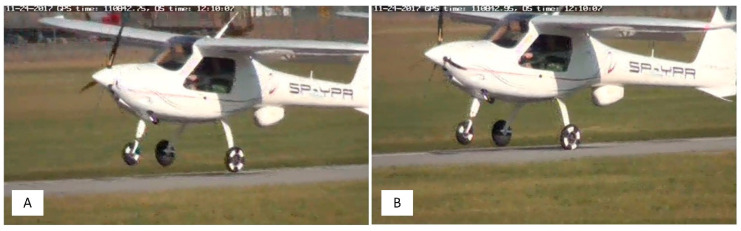
Time-lapse recording of MP-02 touchdown based on the image from the camera and GPS time: just before (**A**) and just after touchdown (**B**).

During the tests, the recorder’s location was as close to the centre of gravity as possible (15 cm in front of the centre of gravity in the longitudinal axis, as shown in Figure 11A). The aircraft also had white markers on the tires so that it is easier to see when the wheels touch the ground (Figure 12). The exact moment of touchdown was identified by video analysis (the image in the ground station was synchronized with GPS time as well as the onboard recorder). White markers were applied to the tires in order to facilitate the assessment of the time when the wheels came into contact with the ground. The obtained contact time determines the moment “0” for the data from the onboard recorder (Figure 12B). For this purpose, a precise GPS receiver was used, updating the navigation message with a frequency of 20 Hz. The markers of the current GPS time and the system time of the ground recorder (rugged mobile computer) are applied to individual video frames obtained from the video camera (Figure 11B). This solution makes it possible to determine the characteristic phenomena occurring during the landing of an aircraft with an accuracy of up to 0.05 s.

### 3.2. Selection and Accuracy of the MEMS Sensors Used

The sensors for the I-31T aircraft were selected due to other experimental studies that were underway, before the authors took up the topic of virtual touchdown detection. Verification was carried out on the MP-02 aircraft, on which accelerometers with a frequency of 1 kHz were intentionally installed. The MEMS class of sensors was selected, as they are very often used in modern integrated systems for indicating and recording flight parameters. In the future, this may allow the method to be widely used in practice.

In order to check measurement uncertainty of the sensors, root mean square (RMS) values were calculated for recordings of 10^6^ samples in static state. Results obtained for accelerometers installed in axes x and z are respectively:

- 0.0019 and 0.0052 for I-31T measurement system (sampling frequency of 50 Hz),
- 0.0028 and 0.0041 for PCIM-01 inertial measurement unit (sampling frequency of 1000 Hz).

**Figure 13:**
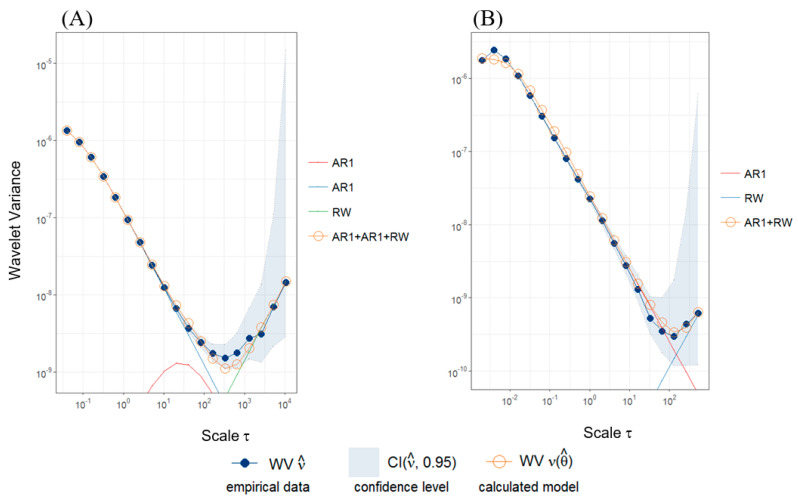
Wavelet variance calculated for 10^6^ observations of a_x_: measurement system installed on the board of I-31T (**A**), PCIM-01 inertial measurement unit (**B**).

Stochastic characteristics of sensors were also investigated using Allan variance [17] and generalized method of wavelet moments (GMWM) [12,26]. Figure 13 presents empirical wavelet variance (WV) of measured data (10^6^ observations per parameter). The GMWM results allowed estimating parameters of process noise which were compared with wavelet variance values for optimized sensor models and real sensors. Plots presented in Figure 13 allow us to understand what kind of processes are contributing to the overall error model. Error model of accelerometer X installed on MP-02 can be specified using a combination of first-order autoregressive process (AR1) and random walk process (RW). Accelerometer X installed onboard the I-31T is characterized by an additional second-order autoregressive process.

### 3.3. Analysis of Sample Data

**Figure 14:**
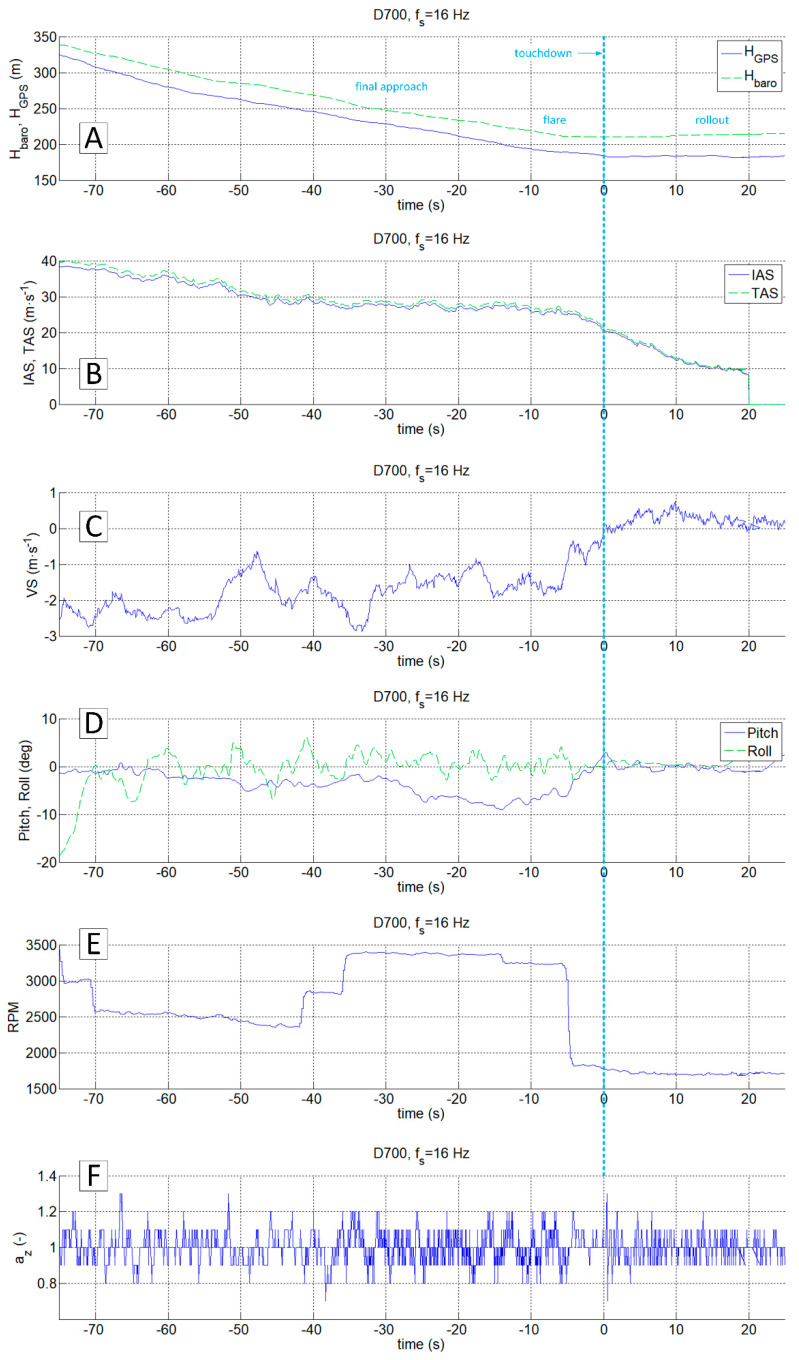
Flight parameters recorded during MP-02 landing No. 4 (12 July 2017, paved runway); recorder D700, f_s_ = 16 Hz; H_baro_ and H_GPS_ (**A**), IAS and TAS (**B**), VS (**C**), Pitch and Roll (**D**), RPM (**E**), a_z_ (**F**).

**Figure 15:**
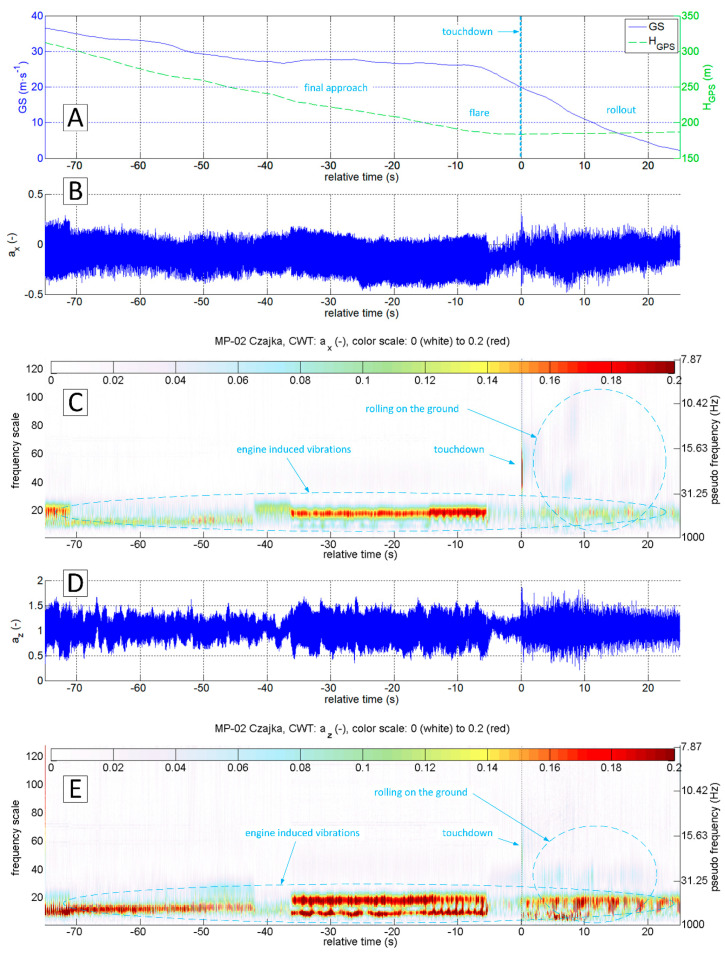
Flight parameters GS (**A**), a_x_ (**B**) and a_z_ (**D**) recorded during MP-02 landing No. 4 (12 July 2017, paved runway) and continuous wavelet transform of the acceleration values a_x_ (**C**) and a_z_ (**E**); PCDL-01 recorder; f_s_ = 1 kHz.

By analysing the data set recorded by the D700 system (Figure 14), it is possible to identify the touchdown time on the $a_{z}$ plot (0 s of relative time). The changes in vertical velocity VS (Figure 14C) play a secondary role in this analysis due to the delays resulting from the design of the aircraft variometer (the process of pressure equalisation by capillary tube in a classic mechanical variometer or algorithms using integrated pressure and inertial measurements in electronic variometers). Information about barometric altitude, GPS altitude (Figure 14A), Euler angles (Figure 14D) as well as TAS and IAS speeds (Figure 14B) are of secondary importance in the process of identifying the touchdown moment. The engine revolutions per minute, also shown in Figure 14E, can be helpful in the analysis of $a_{z}$ acceleration. It enables distinguishing of structural vibrations caused by the engine from the ones coming from external forces (e.g., ground reaction, atmospheric gusts). A rough comparison of engine revs recorded by the D700 with the results of continuous wavelet analysis of $a_{x}$ and $a_{z}$ accelerations from the PCIM-01 device (Figure 15) highlights potential possibilities in using correlated data sets from these two systems.

**Figure 16:**
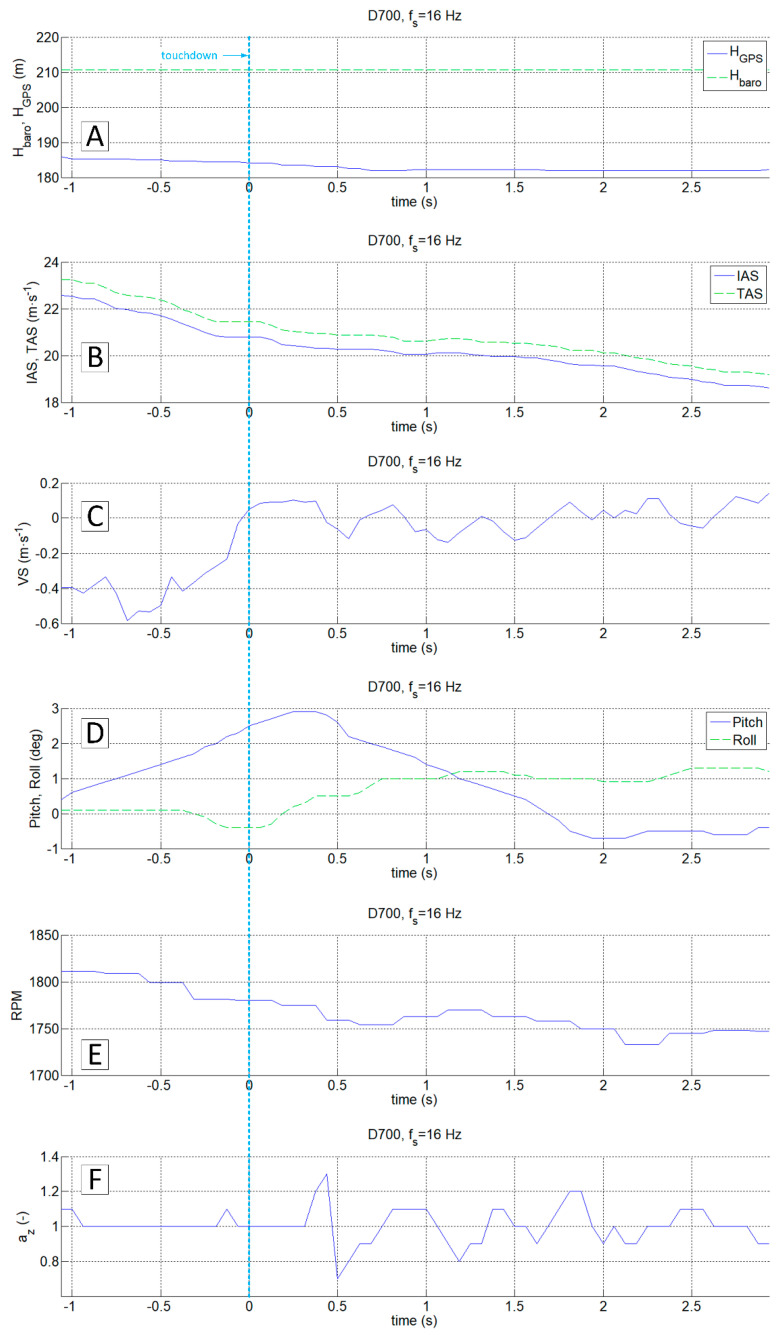
Touch and go MP-02 landing No. 4 (12 July 2017, paved runway); recorder D700, f_s_ = 16 Hz; H_baro_ and H_GPS_ (**A**), IAS and TAS (**B**), VS (**C**), Pitch and Roll (**D**), RPM (**E**), a_z_ (**F**).

**Figure 17:**
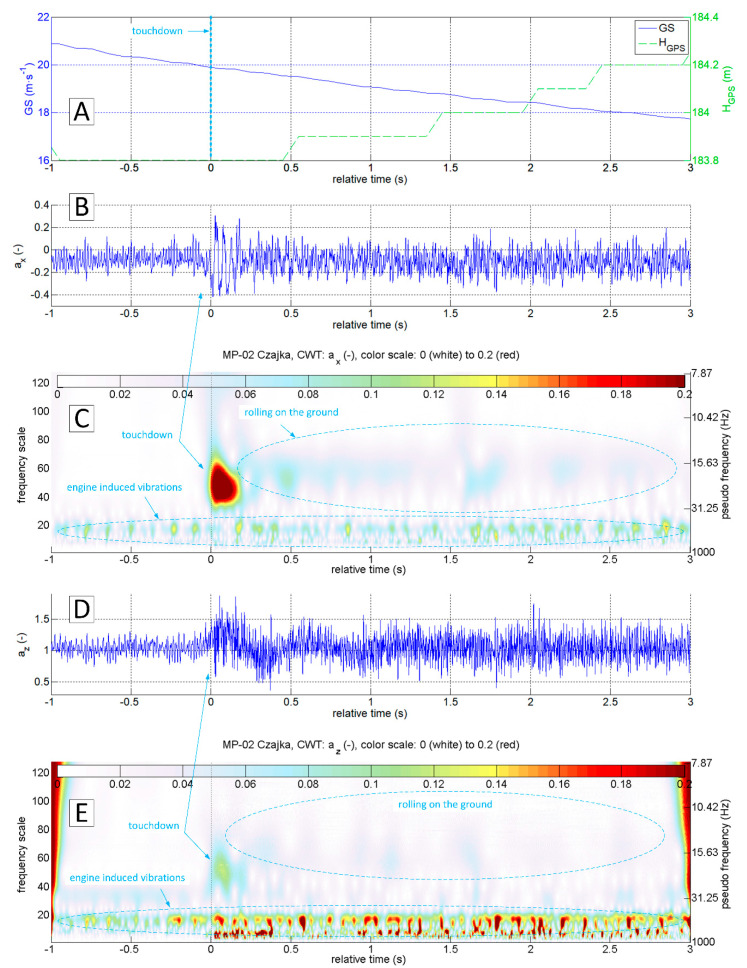
Flight parameters GS (**A**), a_x_ (**B**) and a_z_ (**D**) and CWT of a_x_ (**C**) and a_z_ (**E**) (MP-02 landing No.4, 12 July 2017, paved runway).

The accelerations spectrum subjected to wavelet analysis has a lower limit of 7 Hz (Figure 15C,E). Below this value, there are no significant phenomena in the touchdown detection process, and the slow-changing phenomena associated with aircraft motion are not intense. The moment of a touchdown on the concrete runway is visible on both time series (Figure 15B,D) as well as on charts showing the modified wavelet coefficients of transformations of $a_{x}$ (Figure 15C) and $a_{z}$ (Figure 15E). This event corresponds to the value of $a_{x}$ amplitude exceeding 0.2 (interpreting CWT results), with the maximum frequencies in the range of 15–30 Hz. A detailed analysis of $a_{x}$ acceleration time series in the vicinity of the touchdown point (−1 s to 3 s relative time, Figure 16 and Figure 17) show that $a_{x}$ oscillations reach an amplitude close to 0.3, while the $a_{z}$ amplitude exceeds 0.5 in the 0–0.4 s period. Vibrations with frequencies exceeding 30 Hz are associated with the engine. The CWT plot of $a_{x}$ demonstrates the ground reaction with a mid-frequency of about 10–15 Hz from 1st to 10th second of relative time (Figure 15C). Beyond this point, the aeroplane speed decreased during the roll-out and the recorded vibrations transmitted through the fuselage to the measurement system were extinguished.

In order to compare the data from a classic flight recorder operating at 16 Hz (D700) and a high-frequency recorder, the data from both devices in the vicinity of the touchdown point were presented (Figure 16 and Figure 17, respectively). The observed changes of aircraft height and speed in both cases do not even allow determining of the approximate touchdown point. The plot of the vertical speed on the standard avionics equipment suggests that the touchdown could occur just before or after 1 s or more (VS parameter oscillates around 0). Interpretation of changes in a pitch angle brought similar conclusions. Both signals are not reliable enough to predict the touchdown moment.

The analysis of the vertical acceleration component $a_{z}$ allows identifying of the touchdown with an accuracy of 0.2 s (referring to the time series shown in Figure 16F) and with an accuracy exceeding 0.05 s (Figure 17D). In this particular case, both signals $a_{x}$ and $a_{z}$ carry sufficient information for unambiguous detection of the event. However, the observed phenomenon in the case of $a_{x}$ is much more intense. Both components, $a_{x}$ and $a_{z}$, were recorded at the frequency of 1 kHz with a similar resolution in the time domain.

**Figure 18:**
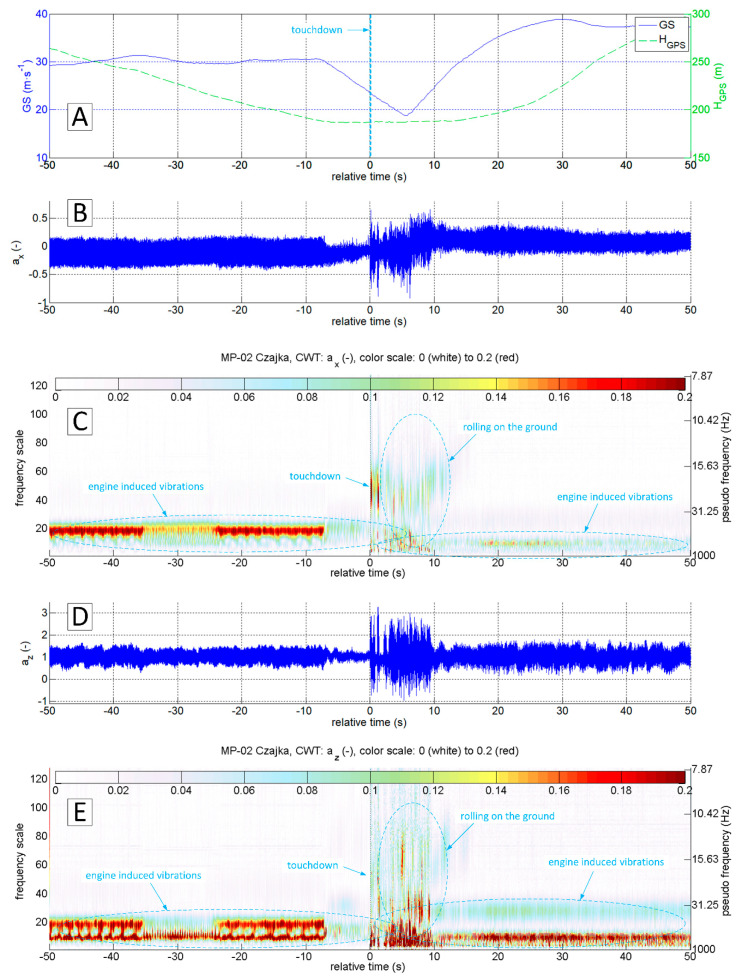
Flight parameters GS (**A**), a_x_ (**B**) and a_z_ (**D**) recorded during MP-02 landing No. 3 and take-off (12 July 2017, unpaved runway 26 EPRJ) and continuous wavelet transform of the a_x_ (**C**) and a_z_ (**E**); PCDL-01 recorder; f_s_ = 1 kHz.

**Figure 19:**
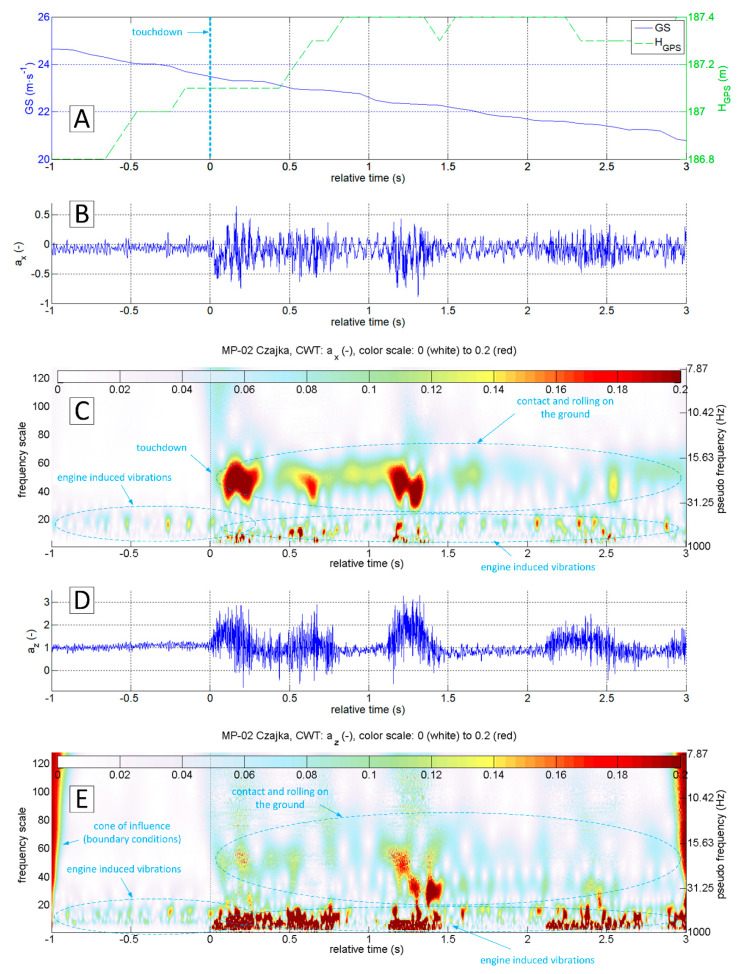
Flight parameters GS (**A**), a_x_ (**B**) and a_z_ (**D**) recorded during MP-02 landing No. 3 and take-off (12 July 2017, unpaved runway 26 EPRJ) and continuous wavelet transform of the a_x_ (**C**) and a_z_ (**E**); PCDL-01 recorder; f_s_ = 1 kHz.

**Figure 20:**
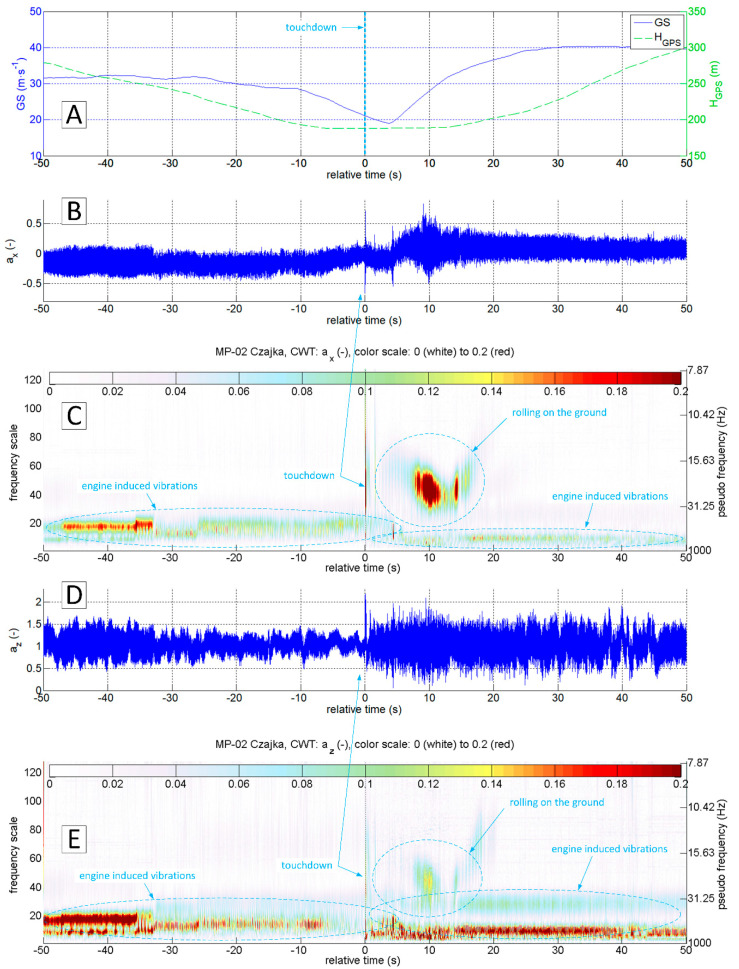
Flight parameters GS (**A**), a_x_ (**B**) and a_z_ (**D**) recorded during MP-02 landing No. 2 and take-off (12 July 2017, paved runway) and continuous wavelet transform of the a_x_ (**C**) and a_z_ (**E**); PCDL-01 recorder; f_s_ = 1 kHz.

**Figure 21:**
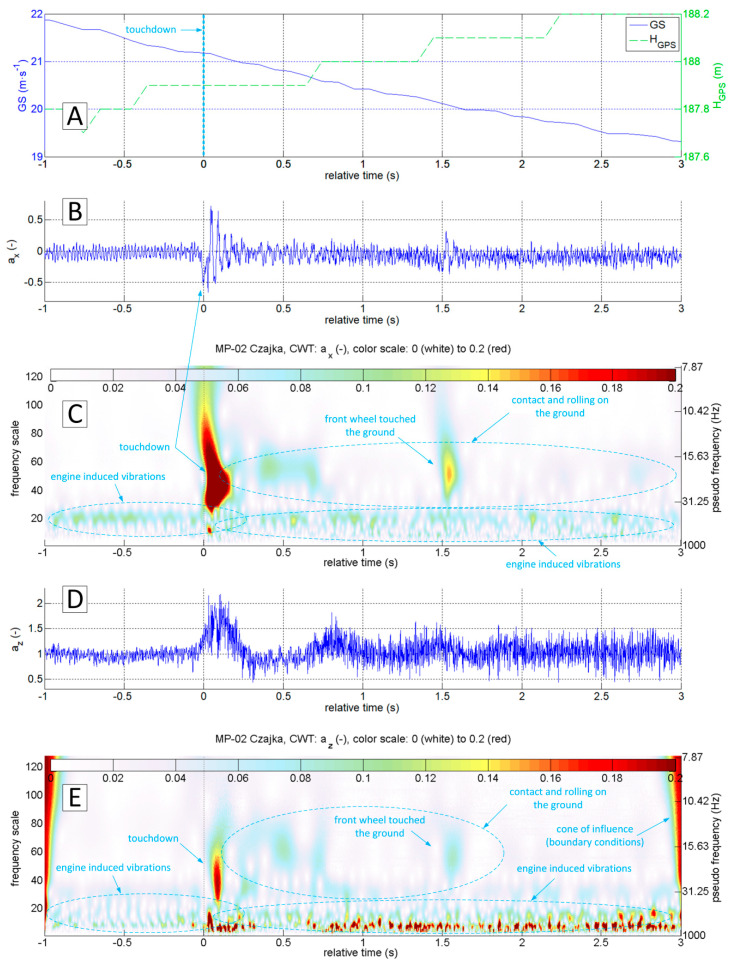
Flight parameters GS (**A**), a_x_ (**B**) and a_z_ (**D**) recorded during MP-02 landing No. 2 and take-off (12 July 2017, paved runway 26 EPRJ) and continuous wavelet transform of the a_x_ (**C**) and a_z_ (**E**); PCDL-01 recorder; f_s_ = 1 kHz.

Figure 18, Figure 19, Figure 20 and Figure 21 present data recorded during two landings followed by immediate take-offs (so-called touch and goes). Figure 18 and Figure 19 refer to a landing on a grass runway. Figure 18 shows data 50 s ahead and 50 s after the touchdown point. The CWT allowed isolating vibrations coming from the propulsion unit and ground reactions. In this particular case, ground reaction, associated with the touchdown of the main gear, is strongly manifested. Therefore, touchdown moment is easy to identify at time series as well as the results of $a_{x}$ and $a_{z}$ wavelet analysis.

Figure 19 shows a narrow segment of data limited to 4 s (starting at 1 s ahead of touchdown). They can be used to distinguish the touchdown of the main and front gear respectively. CWT analysis of $a_{x}$ brings the most accurate result.

A similar analysis was also carried out for the touch and go on the concrete runway. The diagrams presented in Figure 20 indicate the different nature of vibrations coming from the ground reactions. In particular, the z-axis vibration amplitude is much smaller. However, the touchdown moment is still clearly visible. It is possible to distinguish the touchdown of the main and front gear (Figure 21). In this case, CWT analysis of the $a_{x}$ variable gives the best results. For variable $a_{z}$ it is not possible to capture the touchdown of the front undercarriage. This finding shows the potential for the touchdown detection of every undercarriage leg separately (formulation of specific procedures and criteria goes beyond this study). However, the authors included it in future research.

In addition to qualitative studies that prove the concept, quantitative detection algorithms enable designing post-processing software or onboard hardware. The authors proposed three solutions of virtual signals, which were tested using data from 22 flights of the MP-02 aeroplane.

**Figure 22:**
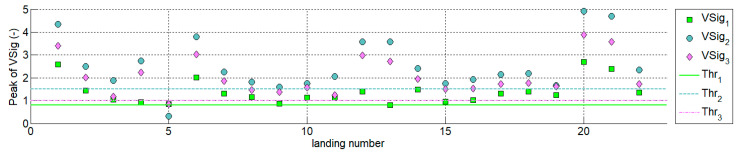
Peak values of VSig_1–3_ determined for 22 MP-02 flights.

The threshold values were determined for MP-02 (for other types this value must be calculated individually). Figure 22 presents the peak values of VSig_1–3_ obtained in the analysis of 22 landings. Table 1 contains information about the detection delay for each of the signals as well as the maximum values of CWT coefficients occurring at the time of the touchdown along with the corresponding frequency scale. One landing only (number 5) was not detectable using all three algorithms. However, analysis of video recording showed that during the touch-and-go only symbolic contact of the left main landing gear tyre with the runway surface occurred. In this case, the touchdown was rudimentary and was not detected (because that is how the thresholds were chosen).

**Figure 23:**
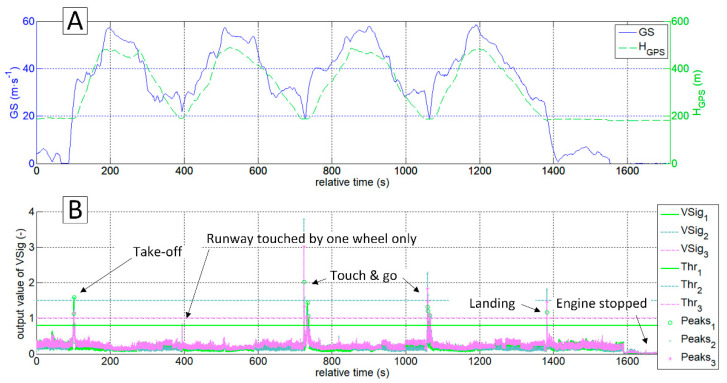
Automatic touchdown detection in four circuit patterns; (**A**) flight parameters and (**B**) virtual signals, detection thresholds and detected peaks.

Figure 23 presents flight speed (GS) and altitude ($H_{\mathrm{GPS}}$) time histories recorded during four circuits performed in a sequence during one flight. They were compared with the values returned by the VSig_1-3_. The chart (B) shows that for three landings the Vsig_1-3_ outputs exceeded the established thresholds in the vicinity of the touchdown. For the case of an aborted touch-and-go at about 400 s, these values are slightly lower than the threshold value. This is the case when the left main landing gear tyre only touched the ground. In addition to the landings, one more event exceeded the threshold value—take-off run. However, the developed algorithms can automatically exclude this event from a set of landings. Additional analysis of $a_{x}$ changes in the vicinity of the touchdown point is required in order to obtain reliable automatic interpretation. The $a_{x}$ decreases after the touchdown and during the roll-out, while after lift-off and during the climb its value increases. It is also beneficial in this case to refer to other flight parameters, such as vertical speed, ground speed and pitch rate.

## 4. Conclusions

The presented methodology is based on the analysis of the flight test campaign results. Several different landing cases gave significant sets of data. Although most of the landings recorded resembled typical load distribution patterns, as described in the requirements, there were many which did not bring unambiguous information about the landing process. During the tests, it turned out that the situation occurs when the signal from the accelerometer installed in the vertical axis of the aircraft (z-axis) is not a sufficient source of information to detect the touchdown of a light aircraft. The results were in line with both expectations and previous practical experiences. This situation may occur if the touchdown is very smooth and delicate, without an unmistakable ascent of the main landing gear impact on the runway surface. The algorithm of the virtual touchdown sensor, using data from a three-axis accelerometer, mandatorily takes into account the analysis of vibration frequencies in the longitudinal axis of the aircraft. Recording of the specific level of vibration amplitude in the predefined frequency range may define a prerequisite for touchdown flagging. Continuous wavelet transformation proved to be helpful in the process of analysing airframe vibrations. It allows precise locating of oscillatory phenomena in the time domain. In the CWT analysis presented in this paper, the authors decided to use the complex Morlet wavelet, which is very well suited for the analysis of impact phenomena. With information about the aeroplane speed as well as the altitude above ground level (AGL), one can impose additional conditions for the virtual touchdown sensor, thus eliminating any possible false alarms coming from in-flight phases other than landing.

The analyses used data recorded during eighteen landings of the I-31T turboprop light aeroplane, twenty-two of the MP-02 ultralight aeroplane, as well as during eighteen experimental flights of the PW-6U glider (archival data from the Advanced In-flight Measurements—AIM2 project [27], not presented in this work). In each case, unambiguous touchdown detection was possible based on the results of CWT analysis of the $a_{x}\;$ acceleration, as the primary source.

The proposed method may be particularly useful for the detection of aircraft touchdown (both in time and space) during off-line bulk data analysis. Preliminary results indicate that on this basis, it is also possible to develop an onboard virtual sensor for touchdown detection, although the authors are aware of the limitations. In order to detect vibrations at specific frequencies, it is necessary to collect an appropriate number of samples, which in turn translates into delays. In the case of the proposed algorithms, using 256 scale levels and with a sampling frequency of 1000 Hz, the detection delay can reach 0.25 s. Taking into account other flight parameters, it is possible to reduce the virtual signal algorithm to 32 scale levels, which will allow a possible reduction to 0.03 s. It should also be remembered that we analyse vibrations recorded by the measuring system attached to the structure of the aircraft fuselage, not vibrations within the landing gear. This results in additional delays in the virtual sensor system.

The main goal of this work was not to develop a solution that could compete with classical measuring systems. The authors, however, intended to create an algorithm for a virtual sensor that would enable accurate detection of the time and place of touchdown, based on the recorded typical set of flight parameters. This goal was achieved with regard to the off-line analysis. In the future, building a test device is planned that will be able detection of selected events, including touchdown, in a real physical system.

## Figures and Tables

**Table 1:** The coefficients calculated using detection algorithms in 22 landings of MP-02 aeroplane.

No.	Landing of MP-02 (GPS Time)	Max. Value of CWT Coeff. (Freq. Scale)	VSig_1_ Peak /Delay	VSig_2_ Peak /Delay	VSig_3_ Peak /Delay	Remarks
1	17 May 2017 #1 10:48:31.9	0.60 (255)	2.58/0.09 s	4.34/0.07 s	3.41/0.06 s	T&G, concrete 26 EPRJ
2	17 May 2017 #2 10:54:21	0.30 (55)	1.45/0.05 s	2.49/0.05 s	2.02/0.05 s	T&G, concrete 26 EPRJ
3	17 May 2017 #3 11:00:39.7	0.20 (249)	1.04/0.10 s	1.86/0.09 s	1.18/0.07 s	T&G, grass 26 EPRJ
4	17 May 2017 #4 11:05:14.5	0.20 (255)	0.93/0.08 s	2.75/0.06 s	2.24/0.06 s	T&G, concrete 26 EPRJ
5	12 July 2017 #1 11:59:46.7	0.70 (24)	0.84/0.09 s	0.32/0.09 s	0.85/0.09 s	T&G, concrete 26 * EPRJ
6	12 July 2017 #2 12:05:15.9	0.45 (184)	2.02/0.04 s	3.79/0.05 s	3.02/0.00 s	T&G, concrete 26 EPRJ
7	12 July 2017 #3 12:10:50.2	0.32 (255)	1.32/0.16 s	2.25/0.10 s	1.84/0.39 s	T&G, grass 26 EPRJ
8	12 July 2017 #4 12:16:13.5	0.27 (140)	1.15/0.05 s	1.82/0.00 s	1.46/0.00 s	LDG, concrete 26 EPRJ
9	12 July 2017 #5 13:23:22.2	0.18 (254)	0.88/0.04 s	1.60/0.04 s	1.38/0.00 s	T&G, concrete 26 EPRJ
10	12 July 2017 #6 13:53:26.3	0.25 (56)	1.13/0.03 s	1.74/0.02 s	1.57/0.03 s	LDG, concrete 26 EPRJ
11	24 November 2017 #1 12:28:13.1	0.22 (100)	1.14/0.06 s	2.07/0.05 s	1.24/0.06 s	T&G, grass 26 EPRJ
12	24 November 2017 #2 12:33:41.4	0.22 (50)	1.40/0.01 s	3.58/0.09 s	2.99/0.09 s	T&G, grass 26 EPRJ
13	24 November 2017 #3 12:39:29.9	0.18 (235)	0.81/0.02 s	3.59/0.04 s	2.72/0.04 s	T&G, grass 26 EPRJ
14	24 Nov 2017 #4 12:45:05.7	0.35 (48)	1.48/0.05 s	2.41/0.09 s	1.94/0.09 s	T&G, grass 26 EPRJ
15	24th November 2017 #5 12:50:52.1	0.21 (30)	0.95/0.09 s	1.74/0.07 s	1.51/0.02 s	T&G, grass 26 EPRJ
16	24 November 2017 #6 12:55:19.6	0.24 (40)	1.02/0.04 s	1.92/0.02 s	1.54/0.02 s	LDG, concrete 26 EPRJ
17	11 May 2018 #1 08:42:58.3	0.30 (56)	1.30/0.03 s	2.15/0.02 s	1.72/0.02 s	T&G, concrete 08 EPRJ
18	11 May 2018 #2 08:48:13.3	0.35 (52)	1.4/0.03 s	2.19/0.02 s	1.74/0.04 s	T&G, concrete 26 EPRJ
19	11 May 2018 #3 09:10:05.3	0.25 (52)	1.25/0.01 s	1.65/0.01 s	1.63/0.01 s	LDG, Laszki 77
20	27 July 2018 #1 14:56:53.6	0.70 (256)	2.70/0.02 s	4.92/0.01 s	3.89/0.01 s	T&G, concrete 08 EPRJ
21	27 July 2018 #2 15:02:11.9	0.55 (256)	2.40/0.04 s	4.71/0.02 s	3.59/0.04 s	T&G, concrete 08 EPRJ
22	27 July 2018 #3 15:07:12.1	0.30 (108)	1.35/0.07 s	2.36/0.03 s	1.72/0.04 s	LDG, concrete 08 EPRJ

*—runway touched by left main wheel only, LDG—landing, T&G—touch and go, EPRJ—Rzeszów-Jasionka airport code.

## Abbreviations

Θ	pitch Euler angle (deg);
Φ	roll Euler angle (deg);
Ψ	yaw Euler angle (deg);
$\lambda_{\mathrm{GPS}}$	GPS longitude (deg);
$\phi_{\mathrm{GPS}}$	GPS latitude (deg);
σ_n_	standard deviation of virtual signal noise;
Ψ(f)	Fourier transform of ψ(t);
ψ(t)	mother wavelet;
$\psi_{\mathrm{ab}}{}^{*}$	basic wavelet;
a	scale factor;
$a_{x}$	longitudinal acceleration related to g_0_;
$a_{y}$	lateral acceleration related to g_0_;
$a_{z}$	vertical acceleration related to g_0_;
A	virtual signal peak
b	wavelet shift;
f(t)	generic function;
f_s_	sampling time (s);
g_0_	standard acceleration due to gravity (9.80665 m·s^−2^);
GS	ground speed (m·s^−1^);
$H_{\mathrm{GPS}}$	GPS altitude (m);
IAS	indicated airspeed (m·s^−1^);
p	roll angular velocity (deg s^−1^);
$p_{s}$	static atmospheric pressure (Pa);
q	pitch angular velocity (deg s^−1^);
r	yaw angular velocity (deg s^−1^);
SNR	signal to noise ratio;
t	time (s);
TAS	true airspeed (m·s^−1^);
TT	true track (m·s^−1^);
VS	vertical speed (m·s^−1^);
